# Size-dependent cytotoxicity of silver nanoparticles to *Azotobacter vinelandii*: Growth inhibition, cell injury, oxidative stress and internalization

**DOI:** 10.1371/journal.pone.0209020

**Published:** 2018-12-19

**Authors:** Li Zhang, Lingli Wu, Youbin Si, Kunhui Shu

**Affiliations:** Anhui Province Key Laboratory of Farmland Ecological Conservation and Pollution Prevention, School of Resources and Environment, Anhui Agricultural University, Hefei, China; Institute of Materials Science, GERMANY

## Abstract

The influence of nanomaterials on the ecological environment is becoming an increasingly hot research field, and many researchers are exploring the mechanisms of nanomaterial toxicity on microorganisms. Herein, we studied the effect of two different sizes of nanosilver (10 nm and 50 nm) on the soil nitrogen fixation by the model bacteria *Azotobacter vinelandii*. Smaller size AgNPs correlated with higher toxicity, which was evident from reduced cell numbers. Flow cytometry analysis further confirmed this finding, which was carried out with the same concentration of 10 mg/L for 12 h, the apoptotic rates were20.23% and 3.14% for 10 nm and 50 nm AgNPs, respectively. Structural damage to cells were obvious under scanning electron microscopy. Nitrogenase activity and gene expression assays revealed that AgNPs could inhibit the nitrogen fixation of *A*. *vinelandii*. The presence of AgNPs caused intracellular reactive oxygen species (ROS) production and electron spin resonance further demonstrated that AgNPs generated hydroxyl radicals, and that AgNPs could cause oxidative damage to bacteria. A combination of Ag content distribution assays and transmission electron microscopy indicated that AgNPs were internalized in *A*. *vinelandii* cells. Overall, this study suggested that the toxicity of AgNPs was size and concentration dependent, and the mechanism of antibacterial effects was determined to involve damage to cell membranes and production of reactive oxygen species leading to enzyme inactivation, gene down-regulation and death by apoptosis.

## Introduction

Nanosilver (AgNPs) is currently the most widely used and fastest growing class of nanomaterials. Due to its unique antibacterial properties, it is widely used in traditional industries and emerging fields such as coatings, food, textiles, cosmetics, water purification, medical treatment [1, 2]. Currently, there are more than 1,600 consumer goods containing nanomaterials, 4.2-fold higher than 380 goods containing AgNPs in 2010 (http://www.nanotechproject.org). The large-scale commercial application of AgNPs has created great economic benefits, but there are also risks of AgNPs entering the environment through various channels [3]. The biological toxicity and impacts of AgNPs on the environment have been a major focus of recent attention.

Previous studies have shown that the microbial toxicity of AgNPs can be caused by inhibition of cell growth, alteration of cell morphology and production of reactive oxygen species (ROS). Beddow et al [4] investigated the effects of AgNPs on different bacteria, including three nitrifying bacteria (*Nitrosomonas europaea*, *Nitrosospira multiformis* and *Nitrosococcus ocean*), and *Escherichia coli*, *Bacillus subtilis*. They found that AgNPs caused significant inhibition to nitrification potential rates (NPRs) in nitrifying bacteria, and adversely affect the growth of *E*. *coli* and *B*. *subtilis*. Bryaskova et al [5] reported that AgNPs have a strong antimicrobial effect against bacteria and fungi. AgNPs have extremely small diameter and large surface area, easily enter into cells or bind to the bacterial surface [6]. Long et al [7] have detected the Ag elements in *E*. *coli* cell membrane and cytoplasm as exposed to AgNPs, and found the cells were severely damaged. Arnaout et al [3] determined that AgNPs cause damage to bacterial cell membranes, leading to leakage of intracellular substances and induction of cell death. Numerous studies have shown that the presence of AgNPs cause the generation of ROS, which can lead to apoptosis, destroy cell membranes, interfere with intracellular antioxidant systems, and damage intracellular components such as DNA [8–10]. Further researches have revealed that AgNPs can enter cells by endocytosis or by other means after contact with bacteria [11], where it can cause cell damage. In summary, after contact with the cells, the AgNPs are firstly adsorbed on the cell surface and then enter into the cell. AgNPs pass through the inner membrane and produce ROS, which leads to arrest of DNA replication, inactivates proteins, and destroys cell membranes. Subsequently the cellular components leak from the damaged cell membranes, and this eventually leads to cell death [12, 13].

The toxicity of AgNPs is determined by many factors, including particle size, type of coating material and morphological structure [14]. Numerous reports have demonstrated that the cytotoxicity of AgNPs is size dependent. Gliga et al [15] proposed that as the size decreases, the specific surface area of AgNPs increases, providing more contact sites for interaction with bacteria. Compared to larger AgNPs, smaller size AgNPs are more likely to cross the cell membrane and enter into the cell, increasing the toxicity to bacteria [16, 17]. Studies have also shown that smaller AgNPs has a larger specific surface area and more active sites, which can produce more ROS and exert stronger antibacterial effects [18, 19].

*Azotobactervinelandii (A*. *vinelandii)* is a gram-negative bacterium belonging to the genus *Azotobacter*, which is easy to grow and fixes nitrogen under aerobic conditions [20]. Soil *Azotobacter* are functional bacteria composed of a variety of bacteria capable of fixing N_2_ in the environment, these including many kinds of nitrogen-fixing forms such as autotrophic nitrogen fixation, symbiotic nitrogen fixation and united nitrogen fixation. Because this type of functional bacterium contains nitrogenase, it can assimilate N_2_ into its own biomass and eventually release it into the soil as part of the soil nitrogen pool [21]. *A*. *vinelandii* can fix nitrogen in the atmosphere, which is also important for studying soil nitrogen cycling.

The aim of this study was to investigate (i) the role of AgNPs size on the growth inhibition of *A*. *vinelandii* (ii) the mechanism of AgNPs toxicity on *A*. *vinelandii*, which was determined by examining cell morphology and apoptosis, nitrogenase activity, gene expression, ROS and ▪OH production (iii) the possibility that the AgNPs are internalized in *A*. *vinelandii* cells. These findings would provide an integrative understanding of nanoparticle-bacterial cell interaction and be of great significance for evaluation of the ecological risk of AgNPs.

## Materials and methods

### Materials and chemicals

AgNPs of two different sizes were used in this study: 10 nm nanosilver (nano-Ag 10, coated with polyvinyl pyrrolidone (PVP)) was purchased from Nanjing Xianfeng Nanomaterial Technology Co. (Nanjing, China); 50 nm nanosilver (nano-Ag 50, without coating agent) was purchased from Nanjing Empero Nanomaterials Co. (Nanjing, China). The purity of the AgNPs were about 99%. The suspensions of AgNPs (1000 mg/L for both sizes) was stored in the dark at 4°C until use.

Annexin V-FITC and PI dye (Annexin V-FITC/PI Apoptosis Dual Stain Kit) was purchased from BD Co. USA. DCFH-DA dye (Reactive Oxygen Species Assay Kit) was purchased from Biyotime Co. China. RNA extraction (UNIQ-10 Column Trizol Total RNA Extraction Kit), reverse transcription (Promega reverse transcription kit) and qPCR reagents (TransStrat Top Green qPCR SuperMix Kit) were purchased from Sangon Biotech Co. Shanghai, Promega Co. Shanghai and TransGen Biotech. Co. Beijing, respectively.

### Characterization of two sizes of AgNPs

Transmission electron microscopy (TEM, H-7650, Hitachi, Japan) were used to determine the primary sizes of the AgNPs at an accelerating voltage of 80kV. Dispersed aqueous solutions of AgNPs were dropped onto a carbon-coated copper grid and dried at room temperature to make TEM samples [22], and at least 5 photographs were taken forobserving. According to the TEM images, the Nano Measure software was used to analyze the particle size of AgNPs to obtain the size distribution. For further characterization of stability of AgNPs, the zeta potential and hydrodynamic size were determined; the AgNPs stock solutions were resuspended in the test media to achieve the desired concentrations (2 mg/L and 10mg/L for nano-Ag 10 and 10mg/L and 100 mg/L for nano-Ag 50, respectively), Dynamic Light Scattering (DLS; Malvern Instrument Zetasizer Nano-ZS90, USA) was used, and samples were incubated at 30°C for 12 h under shaking (220 rpm). For the purpose of silver ion release, suspensions of AgNPs were centrifuged using Amicon Ultra Centrifugal Filters (3 kDa; USA), and the filtrates were measured by inductively coupled plasma mass spectrometry (ICP-MS, 7500CX, Agilent. USA).

### *A*. *vinelandii* culture

The *A*. *vinelandii* was a kind gift from Nanjing Institute of Soil Science, Chinese Academy of Sciences. The *A*. *vinelandii* was grown in 1 L of nitrogen-free medium containing 0.5 g yeast, 20.0 g mannitol, 0.8 g K_2_HPO_4_, 0.2 g KH_2_PO_4_, 0.2 g MgSO_4_·7H_2_O, 0.1 g CaSO_4_·H_2_O, 1 ml trace elements, containing 22 g/L ZnSO_4_·7H_2_O, 5 g/L MnCl_2_·4H_2_O, 5 g/L, FeSO4·7H_2_O, 1.6 g/L CoCl_2_·6H_2_O, 1.6 g/L CuSO_4_·5H_2_O, 7.5 g/L, Na_2_MoO_4_·2H_2_O and 60 g/L EDTA-Na_2_. The culture temperature was maintained at approximately 30°C and the pH remained around 7.2 throughout the experiment. After the bacteria were cultured to logarithmic phase (10^8^ cells/mL), they were collected for the subsequent studies.

### Bacteria growth assays

The *A*. *vinelandii* suspensions were inoculated into solid medium containing two different sizes of AgNPs (nano-Ag 10 and nano-Ag 50) at doses of 1, 2, 5, 10 mg/L, and 1, 10, 50, and 100 mg/L, respectively (Concentration selection came from pre-experiment optimization, which is not shown in this study). At the same time, no AgNPs were added into the control group medium. The bacterial suspension was spread evenly with a sterile inoculating loop. After 30 minutes, the culture dishes were inverted in a 30°C incubator and cultured for 48 hours. The cell numbers were recorded periodically.

### SEM and TEM imaging

The *A*. *vinelandii* were inoculated into culture media containing different doses of AgNPs (2 and 10 mg/L for nano-Ag 10, 10 and 100 mg/L for nano-Ag 50) and incubated for 12 h; the control consisted of *A*. *vinelandii* grown to log phase in culture media without AgNPs. The cells were collected by centrifugation, and all samples were fixed using 2.5% glutaraldehyde, followed by post-fixed in 1% OsO_4_ for 1 h and 3 washes with phosphate buffer saline (PBS, pH 7.2). Finally, samples were subject to gradient dehydration in 30%, 50%, 70%, 80%, 90%, 100% ethanol solution (20 min each time) at room temperature. The processed samples were sectioned, stained with specific dyes and observed under scanning electron microscope and transmission electron microscope (SEM, S-4800, Hitachi, Japan; TEM, H-7650, Hitachi, Japan).

### Flow cytometry (FCM) analysis

Suspensions of *A*. *vinelandii* were inoculated into media containing nano-Ag 10 (2 and 10 mg/L), nano-Ag 50 (10 and 100 mg/L) and medium without AgNPs; cultures were incubated for 12 h. After cultivation, cells were double-labeled with dye and analyzed for apoptosis according to the manufacturer’s protocol. Briefly, the cultured cells were collected by centrifugation and washed with 4°C PBS and centrifuged again. The supernatant was removed, and cells were resuspended in 300 μL diluted Binding Buffer; 5 μL Annexin V-fluorescein isothiocyanate (FITC) was added and samples were incubated in the dark for 15 min. Propidium iodide (PI) dye was added 5 min before the test and 200 μL of Binding Buffer was added. Finally, the apoptosis rate was measured by FACSCalibur flow cytometry (BD Co. USA).

### Nitrogenase activity and gene express studies

Suspensions of *A*. *vinelandii* were inoculated into media containing nano-Ag10 (2 and 10 mg/L), nano-Ag50 (10 and 100 mg/L) and into medium without AgNPs; cultures were incubated for 12 h at 30°C. The nitrogenase activity of *A*. *vinelandii* was determined using the acetylene reduction method [23]. Different *A*. *vinelandii* treatments were introduced into a 15 ml test tube at the same inoculum volume, a 1/10^th^ volume of air was drawn, and an equal volume of 10% acetylene gas was immediately injected, Tubes were sealed with a rubber plug and cultured for another hour. From the test tube, 0.5 mL of gas was injected into a gas chromatograph (7890B, Agilent. USA) to determine the amount of acetylene reduction. The nitrogenase activity of *A*. *vinelandii* was calculated based on the amount of C_2_H_4_ produced / 10^8^ cells.

In this study, the reverse transcription-quantitative polymerase chain reaction (RT-qPCR) technique was used to analyze whether AgNPs were genotoxic to *A*.*vinelandii*. After the 12 h as per previous culture technique, the cells were collected by centrifugation. Approximately 10^8^ cells were placed in a 1.5 mL microcentrifuge tube without RNase. Lysozyme solution (100 μL) was added, and samples were digest at room temperature for 10 min, followed by centrifugation at 12,000×g for 2 min at 4°C. The supernatant was carefully removed and placed into a fresh RNase-free 1.5 mL microcentrifuge tube. Bacterial total RNA was extracted according to the UNIQ-10 Column Trizol Total RNA Extraction Kit procedure. Afterwards, total RNA samples were combined with the random primer in RNase free dH_2_O, heated shock at 70°C for 3min, and immediately placed on ice for 5min. The first strand cDNA was obtained by adding dNTP Mixture, RNase Inhibitor, M-MLV buffer, followed by incubated in a 42°C water bath for 1 h. Primers designed for this study were *nifH*-F (5'-ATCACCGCCATCAACTTCCT-3') and *nifH*-R (5'-GATTTCCTGGGCCTTGTTCTC-3'). Quantitative PCR was performed using a LightCycler 96 PCR system (Roche) in 20 μL of reaction mixture composed of cDNA (2 μL), SYBR Green Master Mix (10 μL), 0.5 μL of each primer, and RNase Free dH_2_O under following condition: 10 min at 95°C, followed by 40 cycles of 30 s at 95°C, 35 s at 61°C. The results of the experiment were calculated using the 2^-ΔΔCt^ method. Nitrogenase activity and gene expression is shown as fold change.

### Reactive oxygen species (ROS) detection and ESR analysis

The presence of AgNPs stimulates production of reactive oxygen species (ROS), which leads to oxidative stress in bacteria. DCFH_2_-DA (2,7-dichlorofluorescein diacetate) can detect ROS, and is able to freely pass through cell membranes to react with intracellular ROS and produce the fluorescent compound dichlorofluorescein (DCF). The level of ROS is determined by detection of DCF fluorescence [24]. In this research, the *A*. *vinelandii* suspensions were separately inoculated into the above AgNPs treatment medium and cells were collected at 0, 2, 4, 6, 12 h, respectively and washed 3 times with PBS buffer. DCFH_2_-DA and nanosilver-free fresh medium were added at a volume ratio of 1:2000 and incubated at 30°C for 30 min. After the incubation, the samples were collected by centrifugation, washed again with PBS and resuspended, and the ROS production in *A*. *vinelandii* cells was measured by Flow Cytometry (BD Co., USA).

All electron spin resonance (ESR) were carried out using a JOEL ESR spectrometer (JES-FA 200, Japan) at room temperature. The acetate buffer solution system pH at 3.6 contained 0.5 mM H_2_O_2_, 50 mM DMPO, different doses of AgNPs and the control contained no AgNPs. A spin trap, DMPO was used to determine that AgNPs can facilitate the generation of hydroxyl radicals (•OH) in the presence of H_2_O_2_. The machine settings are as follows: Field modulation of 1G, microwave power of 20mM and scan range of 100G. The final concentration of each treatment was specified in the figure section.

### Intracellular and membrane Ag contents

The cells were incubated in test media containing nano-Ag 10 (2 and 10 mg/L), nano-Ag 50 (10 and 100 mg/L) for 12 h. Equivalent amounts of bacteria from each sample were lysed via intermittent sonication by an Ultrasonic Cell Crusher (XO-900D, Nanjing) for 1 min, followed by centrifugation at 30,000×g for 30 min. After centrifugation, the supernatant was collected and digested with 2 ml of the mixture (65% HNO3: 30% H_2_O_2_, 1:1) as a cell membrane fraction of *A*. *vinelandii*, and the precipitate containing the cytoplasmic portion was treated in the same manner. ICP-MS (7500CX, Agilent. USA) was then used to measure the Ag content [7].

### Statistical analysis

SPSS19.0 software was used for the differential analysis of the experimental data with the Student’s t-test. There was a significant difference when the P < 0.05.

## Results and discussion

### Characterization of two different sizes of AgNPs

**Fig 1:**
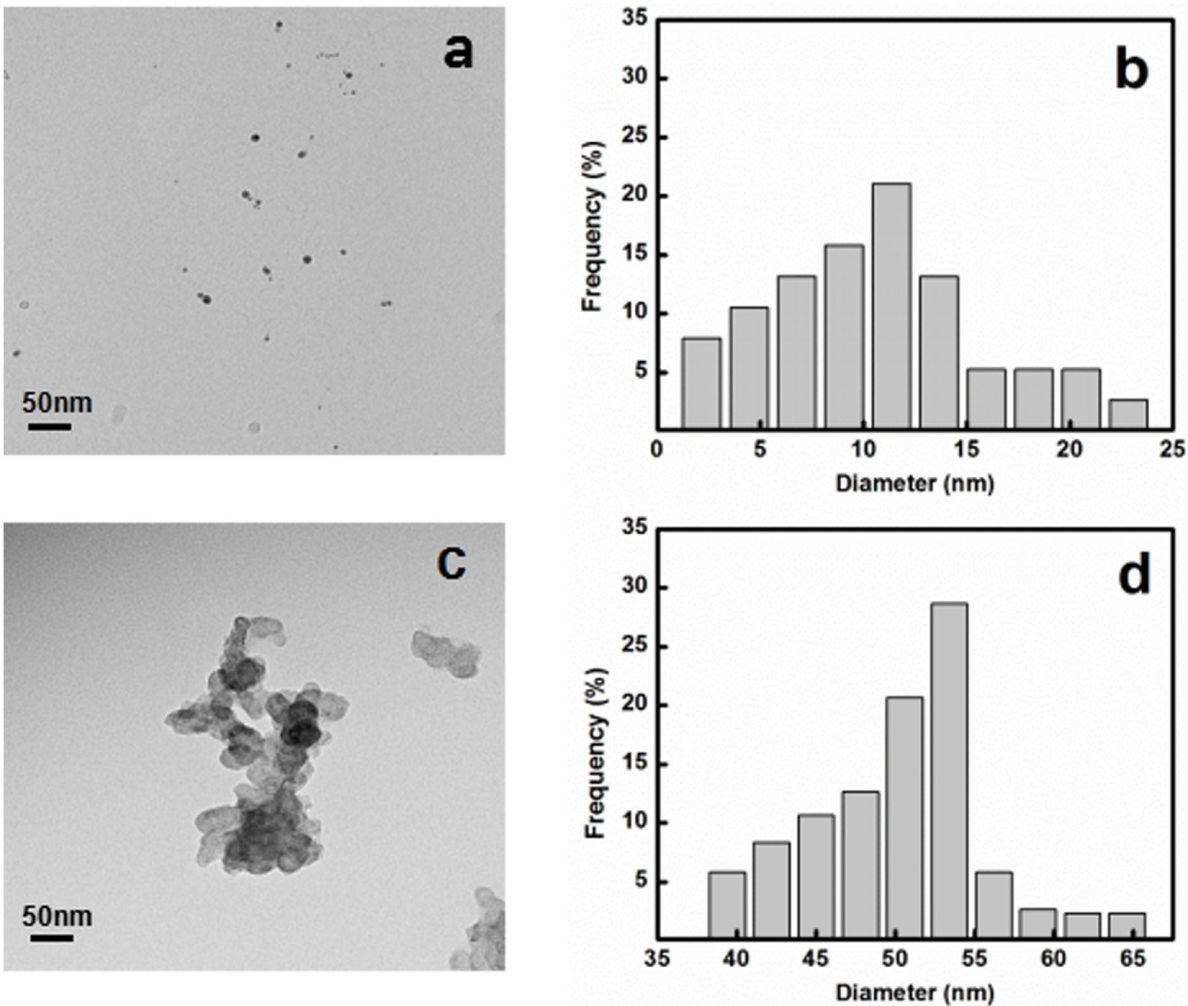
The characterization of different sizes of silver nanoparticles by TEM. TEM images of (a) nano-Ag 10 and (c) nano-Ag 50. Frequency of size distribution for (b) nano-Ag 10 and (d) nano-Ag 50; scale bar: 100nm.

The characterization results of the sizes and properties of supplied nanoparticles are presented in Fig 1. As shown in the TEM image (Fig 1a and 1c), the shapes of AgNPs in this study was relatively uniform, and both were approximately spherical. Under the same magnification, the size of nano-Ag 10 was obviously smaller than nano-Ag 50, and had a better dispersibility. In addition, nano-Ag 50 agglomerated more than nano-Ag10 due to the absence of stabilizer in the solution [25]. The size distribution of two different AgNPs from TEM images was determined and average sizes were about 10.48±0.94 and 48.08±3.44 nm for nano-Ag 10 and nano-Ag 50, respectively, these values were similar to those specified by the manufacturer. Since the characterization between two used concentrations of the two sizes of AgNPs was not significantly different, the highest concentrations data were representative. The Z-average hydrodynamic diameter was 162.9±3.5 nm with polydispersity index (PDI) 0.4±0.02 for nano-Ag 10 and 750.7±65.8 nm with PDI 0.91±0.11 for nano-Ag 50 as monitored by DLS. The surface charges of the both AgNPs were highly negative as indicated by zeta potential values: -22.5±0.11 mV for nano-Ag 10 and -18.1±0.15 mV for nano-Ag 50, respectively (Table 1). As for dissolution, the nano-Ag 10 at the highest working concentration of 10 mg/L was found to contain 0.12% Ag ions, and the nano-Ag 50 at a dose of 100 mg/L contained less than 0.02% release (shown in Table 1), indicating that smaller AgNPs release more Ag in biological medium. The amount of Ag^+^ from both AgNPs were less than 0.5%, which may be due to the agglomeration reduce the surface-to-volume ratio of AgNPs [15]. Because of this, extremely low Ag^+^ release in our research would not be considered in toxicity mechanism of AgNPs. In sum, these results indicated that nano-Ag 10 in tested medium showed a moderate stability and agglomeration while the nano-Ag 50 was less stable.

**Table 1:** Characterization of AgNPs.

	Nano-Ag 10	Nano-Ag 50
Zeta potential (mV)	-22.50±0.11	-18.10±0.15
Polydispersity index (PDI)	0.40±0.02	0.91±0.11
Hydrodynamic diameter (nm)	162.9±3.5	750.7±65.8
Ag released (%)	0.12	0.013

The physicochemical parameters of two sizes of AgNPs were measured using zetasizer after resuspension in test medium for 12h, and the polydisperity index (PDI) was obtained through determining the hydrodynamic diameter. All results were replicated 3 times.

AgNPs had a large specific surface area and high surface potential, thus, they tended to agglomeration, which was affected by various environmental factors such as the coating agent, the type of ions in the solution, the ionic strength, pH [26]. Studies have shown that PVP as a coating material is non-toxic [27], which enhance the dispersability of AgNPs. Several reports have demonstrated that the reduction in toxicity may be attributed to a decrease in release of silver ions and a reduction in surface area when AgNPs form agglomerates [6, 28]. Thus, the characterization of AgNPs may provide a possible reason for differences in antibacterial actions of the two sizes.

### Effect of AgNPs on the growth of *A*. *vinelandii*

**Fig 2:**
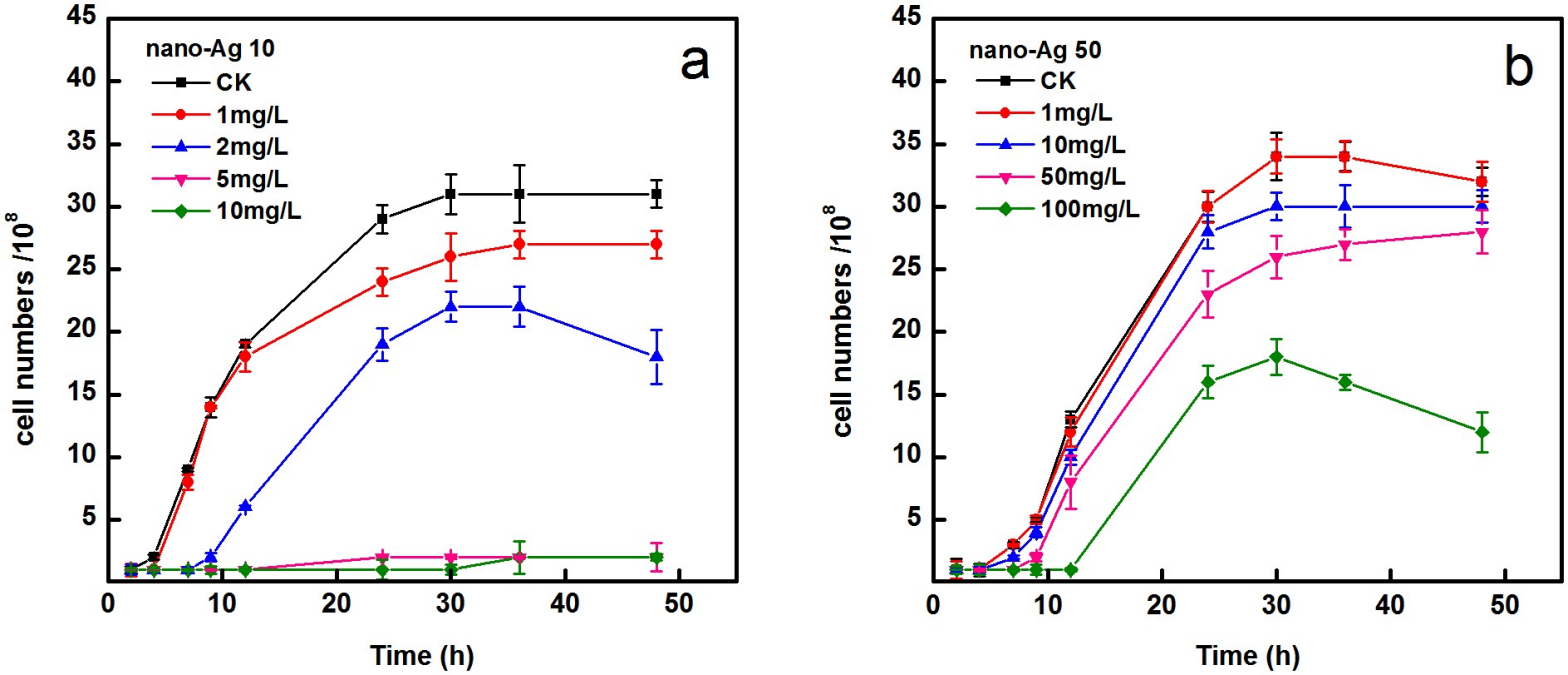
Effect of two different sizes of AgNPs on the growth of *A*. *vinelandii*. (a) nano-Ag 10 and (b) nano-Ag 50. The bacteria were cultured for 48 h in media with and without AgNPs.

Cultures of *A*. *vinelandii* were treated for 48 hours with 2, 5, 10 mg/L of nano-Ag 10 and 10, 50, 100 mg/L of nano-Ag 50, respectively (Fig 2). As shown in the Fig 2b, nano-Ag 50 below 50 mg/L did not produce significant toxicity to cells relative to the control, whereas a treatment of nano-Ag 10 at 5 mg/L resulted in marked inhibition; this suggested that the bactericidal effect of AgNPs may be related to dose and particle size.

Numerous studies have indicated that NPs exhibit strong biotoxicity. Yu et al [22] studied the toxicity of four widely used NPs (TiO_2_, SiO_2_, Ag and CdTe/CdS) to *Chlamydomonas reinhardtii* and found different cytotoxicity effects. Reyes et al [29] revealed that growth rate of *A*. *vinelandii* biofilms was significantly reduced as exposed to CuNPs. Similarly, Gambino et al [30] explored the toxicity of AgNPs (10 nm) on soil bacteria *A*. *vinelandii* and *Bacillus subtilis* (*B*. *subtilis*). The authors found that the 0.1 mg/L of AgNPs significantly inhibited growth of *A*.*vinelandii*; however, the growth rate of *B*. *subtilis* showed a significant decline only when the concentration of AgNPs reached 100 mg/L. It seems that increased AgNPs concentration serve as an effective form of antibacterial action [30]. Our results also provide conclusive evidence for the toxicity of AgNPs is dose-dependent. In addition to concentration, size is also an important parameter for evaluating the biotoxicity of nanomaterials [31]. Previous investigations demonstrated that smaller sizes of AgNPs had higher toxicities [32, 33]. Because smaller AgNPs have higher surface areas and particle numbers per unit mass, the contact area with organisms is expanded and more silver ions are released. Moreover, smaller AgNPs have been shown to penetrate the cell membrane more easily [16, 34]. The present study indicates that the tested AgNPs exert antibacterial activities in both size and concentration dependent manners.

### Effect of AgNPs on cell morphology of *A*. *vinelandii*

**Fig 3:**
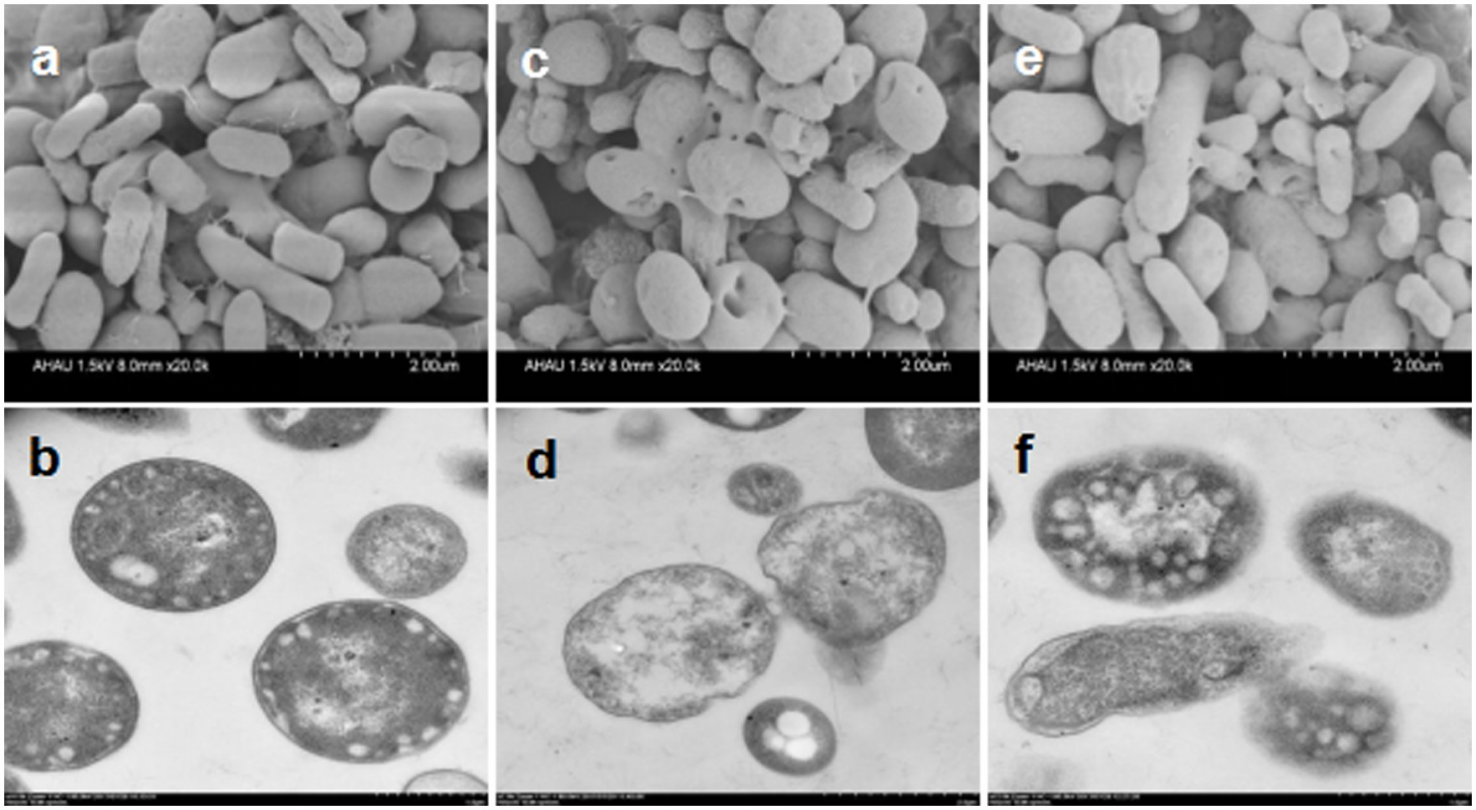
The effect of AgNPs on the cell morphology of *A*. *vinelandii* observed by SEM (upper) and TEM (lower). (a) SEM and (b) TEM images of a control group in which no AgNPs are added; (c) SEM and (d) TEM images of nano-Ag 10 at a concentration of 10 mg/L; (e) SEM and (f) TEM images of nano-Ag 50 at a concentration of 100 mg/L. All treatment groups were exposed for 12 h.

The changes in cell morphology were observed observed with 10 mg/L of nano-Ag10 and 100 mg/L of nano-Ag 50 (Fig 3), which were also the concentrations where an effect on cell growth was noticed. Cells exposed to AgNPs differed significantly from the control. The SEM results showed that there were small holes on the surface of the AgNPs-treated cells, indicating that the bacterial cell surface was damaged after 12 h interaction with AgNPs. Additional TEM observations confirmed this finding, as the figures indicated that the cell membrane edges of *A*. *vinelandii* were rough and fuzzy and there were substances leak inside the cells. These might due to the attachment of AgNPs to the cell membrane surface, disrupting the cell membrane and cell wall. Part of AgNPs may even reach the cytoplasm and intereact with the intracellular components, caused leakage of the contents.

The main components of the cell membrane are proteins and lipids, and AgNPs can interact with proteins to form complexes with substances containing oxygen, phosphorus, sulfur or nitrogen atoms [35, 36]. Studies have shown that the reaction of AgNPs with sulfur-containing membrane proteins may lead to inactivation of membrane-bound enzymes and proteins [14, 35]. AgNPs can also interfere with the respiratory chain and reduce energy production when attacking the membrane [13]. Several investigations have reported that AgNPs could attack unsaturated fatty acids on cell membranes and change the membrane fluidity which can hinder membrane function by destroying membrane permeability and integrity [13, 37]. Thus, researchers have indicated that cells treated with AgNPs are deformed. Michels et al [38]. studied the inhibition of two different nanomaterials (silver and magnetite) on ammonia-oxidizing bacteria and found that the nanomaterials would continue to adsorb on the surface of the bacterial cell membrane until the surface morphology was completely destroyed. This finding was supported by Giao et al [39]. AgNPs caused severe aberration to the cell morphology of ammonia-oxidizing bacteria and could cause bacterial death by damaging the cell membrane. Li et al’s study found that the AgNPs treated *E*. *coli* cells were deformed, with pits and voids on the surface, and some cells also showed a large leakage [37].

In our previous studies, many small pits were found in the cell wall of bacteria which may have been caused by penetration of AgNPs [40]. After passing through the cell wall, AgNPs can accumulate on the surface of the cell membrane, disrupt the cell membrane structureand alter its permeability. AgNPs can also enter into the cell, causing the DNA to become concentrated and stressed, and DNA accumulates in combination with the cytoplasm of the damaged bacteria; this eventually results in leakage of cellular components, leading to bacterial death [18, 41]. In summary, the direct destruction of cell wall and cell membrane by AgNPs may be an important antibacterial mechanism, which needs further study.

### Effect of AgNPs on cell apoptosis of *A*. *vinelandii*

**Fig 4:**
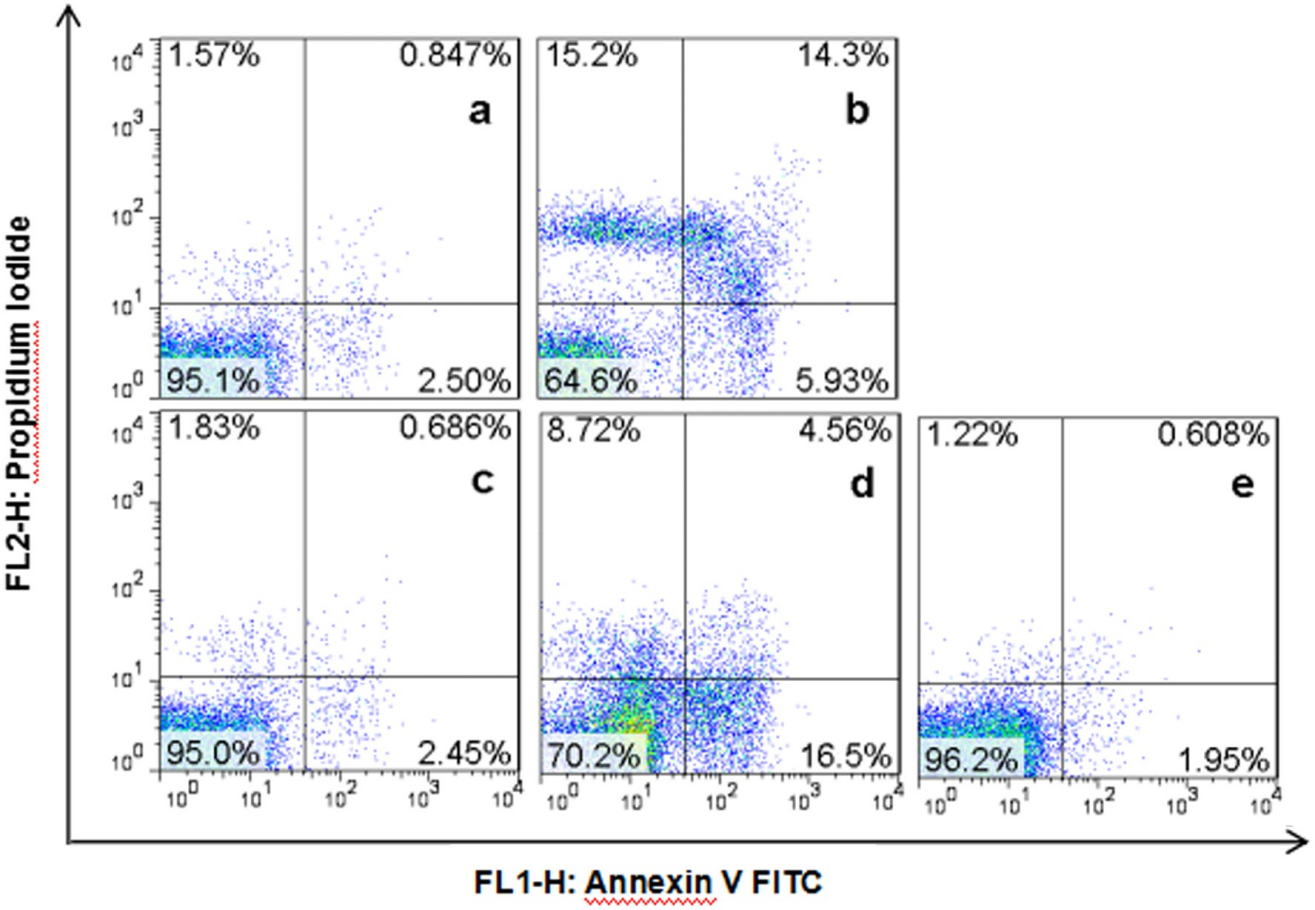
Effect of AgNPs on cell apoptosis of *A*. *vinelandii*. (a) nano-Ag 10 at a concentration of 2 mg/L; (b) nano-Ag 10 at a concentration of 10 mg/L; (c) nano- Ag50 at a concentration of 10 mg/L; (d) nano-Ag 50 at a concentration of 100 mg/L; (e) no AgNPs added. All treatments were exposed for 12 h.

Annexin V is a Ca^+^-dependent protein that binds phosphatidylserine (PS) with high affinity outside the cell membrane to label cells during early-stage apoptosis. PI is a nucleic acid dye that can pass through the membrane of cells in late-stage apoptosis and necrosis to stain the nucleus [34]. After exposured to AgNPs for 12 h, the apoptosis ratios were observed. The apoptosis rate was slightly elevated for *A*.*vinelandii* treated with nano-Ag10 at 2 mg/L (Fig 4a), relative to the control group (3.35% and 2.58%, respectively). The apoptosis rate was 20.23% when when *A*. *vinelandii* were exposed to 10 mg/L of nano-Ag 10 (Fig 4b). However, the apoptosis rate of cell treated with the same concentration of nano-Ag 50 treatment was 3.14% (Fig 4c). Only the concentration was increased to 100 m/L did the apoptosis rate reach 21.06% (Fig 4d). This result is consistent with those of the bacterial growth assays, which indicated that the toxicity of AgNPs to *A*. *vinelandii* significantly increased with decreasing nominal particle size.

Apoptosis is a genetically regulated and orderly death caused by certain physiological or pathological stimulation of cells [42]. A wide variety of compounds, from antibacterial organics to nanoparticles, have been shown to cause bacterial apoptosis [43–45], and our previous research also explored the apoptosis of *Nitrosomonas europaea* under AgNPs exposure [40]. Apoptosis may be present in most bacteria but activated differentially depending on the stimulus [43]. ROS are considered to be a major mechanism of apoptosis in nanoparticles treatments, and DNA damage is a common apoptotic mark [43, 46]. Piao et al [47] reported that the destruction of mitochondrial transmembrane potential is one of the earliest events in the process of apoptosis. Zapór et al [48] confirmed the finding that AgNPs can cause an increase in ROS production to induce mitochondria-mediated apoptosis. Furthermore, AgNPs will cause autophagy function disorder by generating ROS; excessive autophagy can also lead to apoptosis [9]. The apoptotic features in bacteria and eukaryotic cells have been found in high similarity [43], but further research is needed to characterize how ROS induce apoptosis in bacterial cells.

### Effect of AgNPs on nitrogenase activity and *nifH* expression of *A*. *vinelandii*

**Fig 5:**
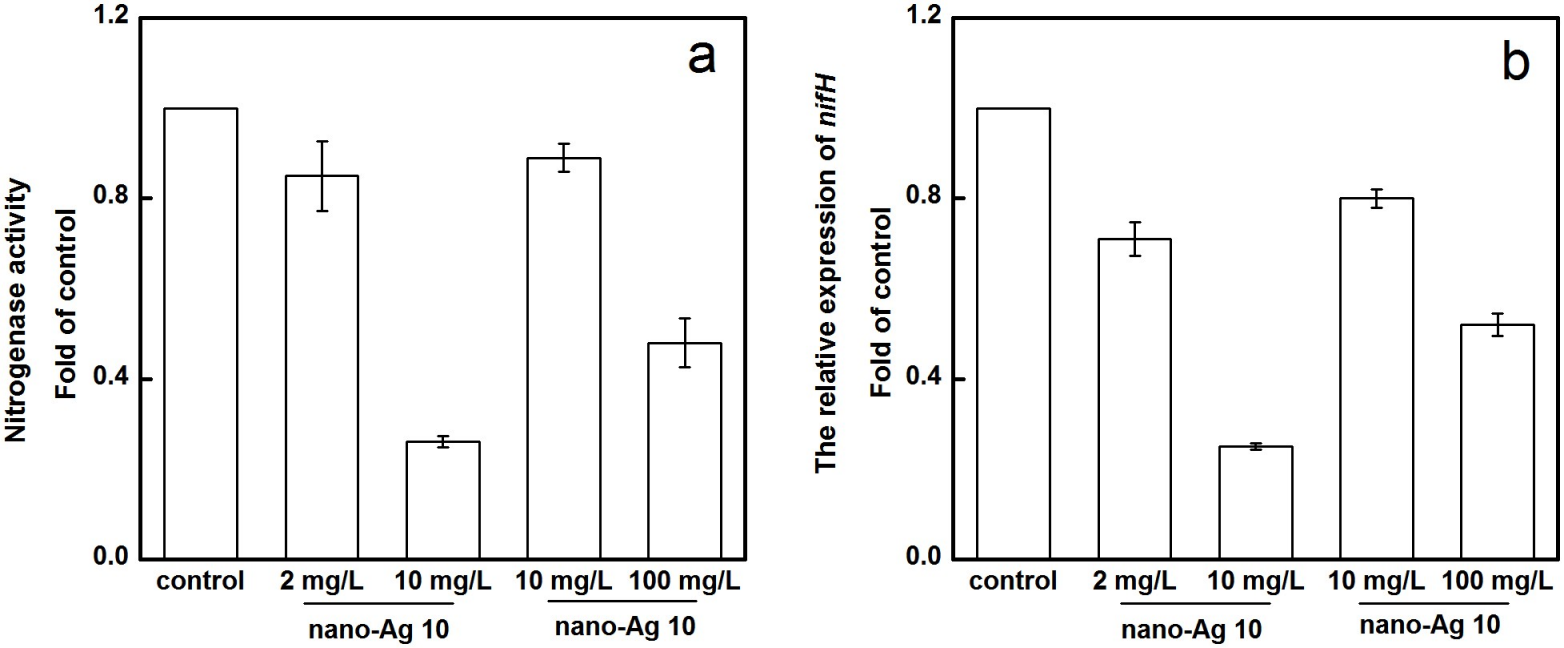
Nitrogenase activity (a) and relative expression of *nifH* (b) of *A*. *vinelandii* exposed to AgNPs for 12 h.

The assay result showed that the nitrogenase activity and expression of the nitrogenase gene *nifH* were significantly inhibited (Fig 5) after exposure of *A*.*vinelandii* to two sizes and different doses of AgNPs for 12 h; the effect of nano-Ag 10 treatment was more obvious. These results were well corresponded with the observations of a reduced effect in apoptosis rates from nano-Ag 50 exposure, nano-Ag 10 has a stronger destructive effect on cells. Our research suggested that AgNPs not only induced cytotoxicity, but also caused genotoxicity and impaired nitrogen fixation in *A*.*vinelandii* cells.

All kinds of nitrogen-fixing organisms are able to fix the molecules of ammonia in the air at room temperature and atmospheric pressure and transform them into combined nitrogen which can be used by organisms; these include symbiotic rhizobia, autogenous, combined nitrogen-fixing bacteria, cyanobacteria, and photosynthetic nitrogen-fixing bacteria. Nitrogen fixation is accomplished by a catalytic protein-nitrogenase enzyme [49]. It has been found that all nitrogen-fixing microorganisms contain the ferritin-encoding *nifH* gene, and the phylogenetic tree of the *nifH* gene is highly consistent with that of 16s RNA [50]. The *nifH* gene is one of the key areas for scientists to study biological nitrogen fixation. Molecular biology analysis based on the *nifH* gene provided a more sensitive and accurate analytical method for the nitrogen fixation than traditional culture.

An important feature of nitrogenase is their high sensitivity to oxygen. The result of gene assay showed that AgNPs inhibited the expression of *nifH* gene, thereby decreasing the activity of nitrogenase. Moreover, exposure of bacteria to AgNPs increased ROS level in bacteria, also affected the activity of nitrogenase. Abd-Alla et al found there was a significant reduction in nitrogenase activity of faba bean *Rhizobium leguminosarum* exposed to AgNPs, and they suggested that presence of AgNPs decreasd the viability of the rhizobial cell [51]. Zarate-Cruz examined the physiological response of *Azolla filiculoides* to the presence two sizes of ZnO NPs (26.7±1 nm and 238±30.7 nm), and found the nitrogenase activity of *Azolla filiculoides* decreased under treatment with all the ZnO NPs concentrations regardless of particle size [52].

### Reactive oxygen species (ROS) generation and free radicals induced by AgNPs

**Fig 6:**
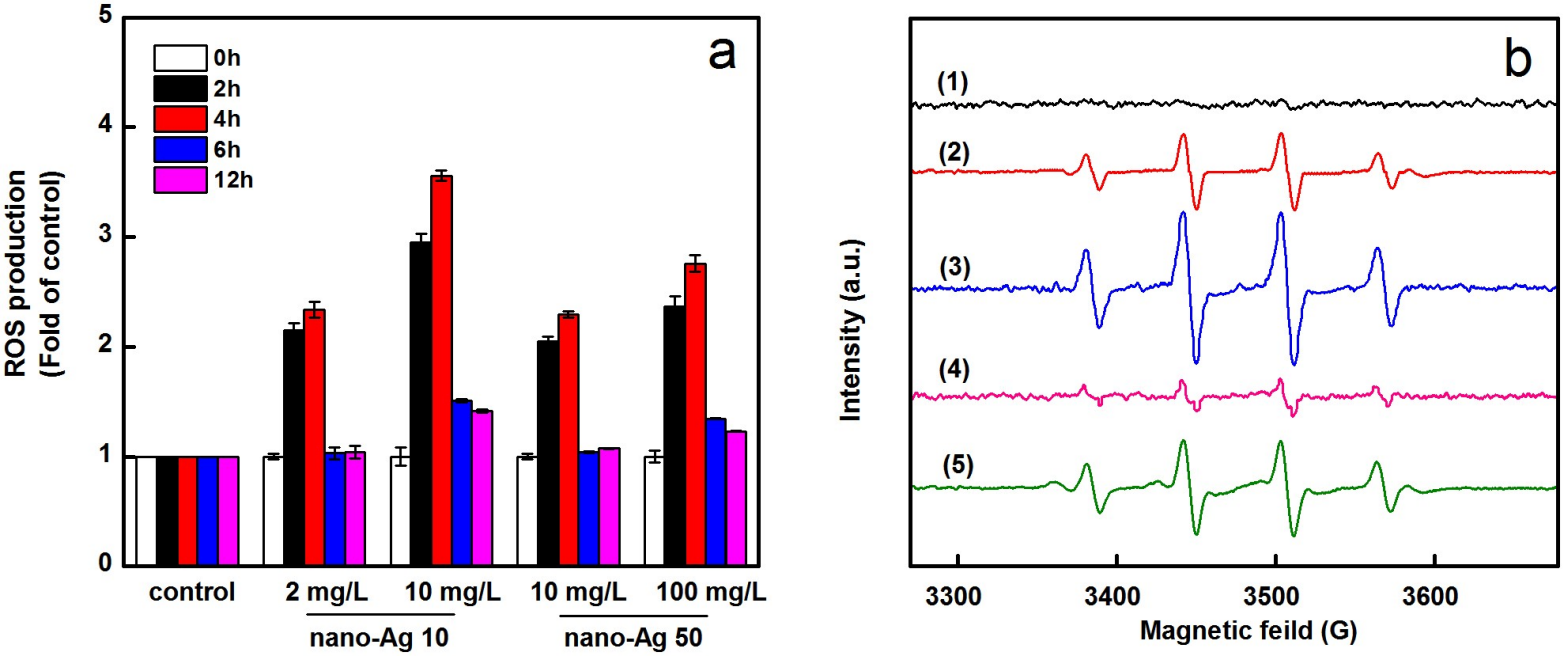
Changes in intracellular ROS levels of *A*.*vinelandii* (a) and generation of hydroxyl radicals by two sizes of AgNPs (b). Electron spin resonance (ESR) spectra were obtained from samples containing 50 mM DMPO + 0.5 mM H_2_O_2_ + 10 mM pH 3.6 HAc-NaAc in the presence of control (1), 2 mg/L (2), 10 mg/L (3) of nano-Ag 10, and 10 mg/L (4), 100 mg/L (5) of nano-Ag 50, respectively.

The reactive oxygen species (ROS) are a type of oxygen-containing highly reactive molecules, including hydroxyl radicals (·OH), superoxide anions (·O_2_^-^), singlet oxygen (^1^O_2_), and hydrogen peroxide (H_2_O_2_) [53]. The generation of ROS is one of the mechanisms by which nanomaterials exert their toxicity. Electron spin resonance spectroscopy is the standard method for quantification of ROS [54]. As shown in Fig 6. Both nano-Ag 10 and nano-Ag 50 (Fig 6a) significantly increased ROS production in *A*. *vinelandii* cells in dose-dependent. It is observed that the amount of ROS produced is higher for nano-Ag 10 than nano-Ag 50. Obviously, the ROS production at 4 h appeared to be greater compared to 6 h which is higher than control. And the reduction of ROS probably due to the ROS generation triggered the antioxidant system in the bacteria cells which decreased ROS levels subsequently [55]. Results for ESR spectra obtained for solutions containing DMPO spin trap without or with AgNPs are shown in Fig 6b. The ESR spectra of the two sizes of AgNPs are consistent with changes in ROS levels. The observation rationalized that nano-Ag 10 has higher antimicrobial activity than nano-Ag 50.

Several previous studies have demonstrated that mechanism by which AgNPs induce hydroxyl radicals in the presence of hydrogen peroxide. The current model involves AgNPs acting as a reagent and generates hydroxyl radicals through a Fenton-like reaction (Ag + H_2_O_2_ + H^+^ = Ag^+^ + •OH + H_2_O) [56, 57]. Thus the level of AgNPs would affect the production of hydroxyl radicals and ROS. This is also consistent with the dose-dependent inhibition on *A*. *vinelandii* growth after AgNPs exposure. Recent studies suggest that ROS generation and oxidative stress are the likely mechanism of AgNPs toxicity [58]. There are antioxidant systems in the cells, which mainly include antioxidant enzymes and antioxidants such as SOD and GSH [59]. Studies have shown that the presence of ROS will activate SOD or GSH to maintain a dynamic balance between oxidation and antioxidant levels [8, 60]. Oxidative stress occurs when ROS generation exceeds the capacity of the antioxidant defense mechanism. ROS and oxidative stress harm cell membrane, leading to lipid peroxidation [48, 61]. In addition, DNA damage, apoptotic cell death, reduction in ATP generation are also induced by ROS [62, 63], and eventually lead to cell death. Thus, it is important to study antibacterial effects of AgNPs and the underlying mechanisms of ROS-mediated damage in AgNPs treated cells should be further explored.

### AgNPs distribution in bacteria

**Fig 7:**
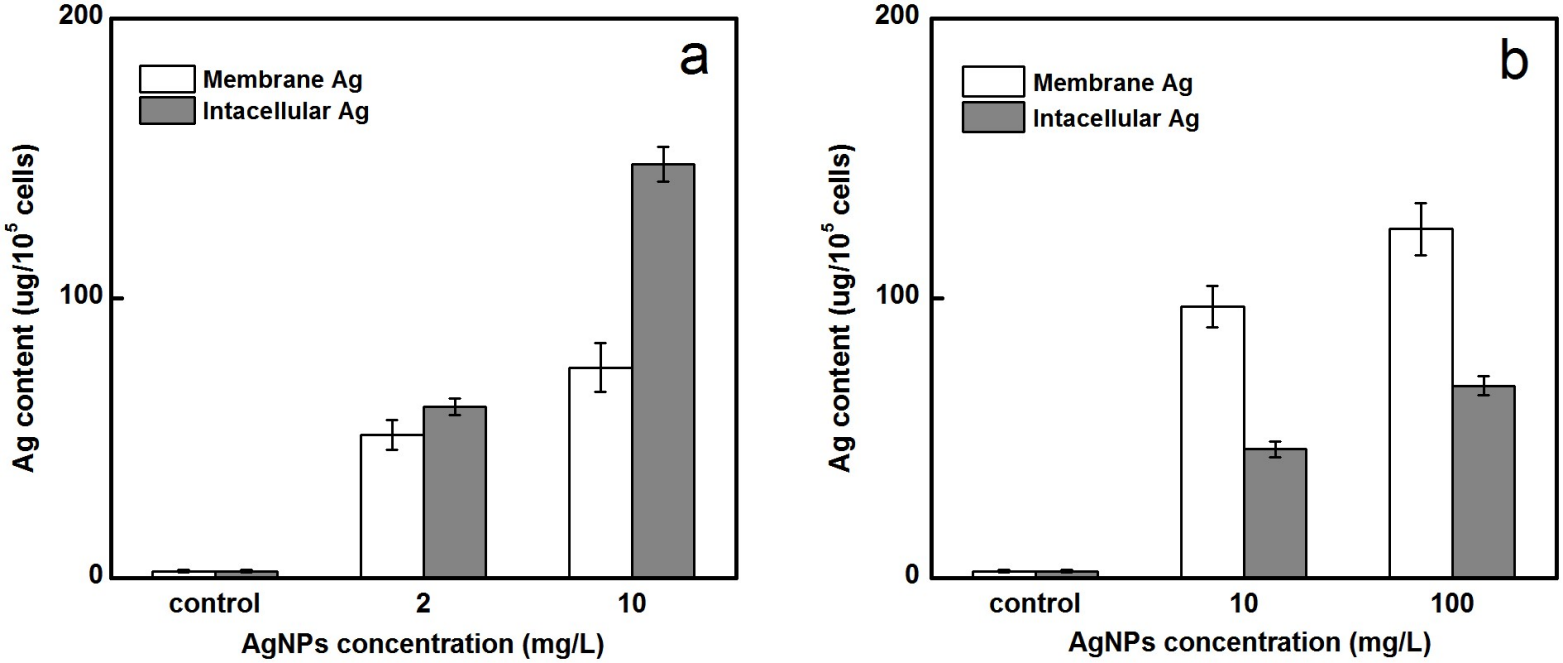
Intracellular and membrane Ag contents of control cells and cells treated with two size of AgNPs for 12h. (a) nano-Ag 10 and (b) nano-Ag 50.

The intracellular and cell-wall-bound Ag contents of *A*. *vinelandii* were determined after the cells were treated with two different sizes of AgNPs for 12h (Fig 7). Compared with the treatment group, the control group cells had almost no AgNPs present, which further demonstrated the internalization of AgNPs in *A*. *vinelandii*. In addition, the intracellular Ag content in the nano-Ag 10 (Fig 7a) treatment group was significantly higher than that on the cell membrane, whereas the nano-Ag 50 (Fig 7b) treatment group had the opposite result, with most of the Ag on the cell membrane. This phenomenon was attributed to that smaller AgNPs entered into cell more easily [16, 64].

Similarly, Long et al [7] observed that the amounts of cytoplasmic Ag were slightly higher than membrane Ag, which proved that regardless of the form (particles or ions) of AgNPs, it can enter into the cells. Thus, they concluded that the AgNPs exert antibacterial activities though the combination of internal and external methods; Ag could bind to the cell membrane and destroy the respiratory chain protein, or it could enter into the cells to cause bacterial damage. Uptake of AgNPs has been investigated in different cells and found to occur through the following steps: initially, they bind on the cell membrane, with the internalization occurring secondly [65]. Many analyses using flow cytometry and electron microscopy showed that AgNPs could enter into cells by penetration or phagocytosis [62, 66]. Studies suggested that the internalization of Ag might exert toxicity on cell membranes and cytoplasm through a Trojan horse type mechanism, which explains the release of Ag ions from AgNPs [17, 48, 67].

Combining Ag distribution results with previous electron microscopy images showed that smaller Ag nanoparticles with higher surface areas could damage cell membranes by direct interaction with cell membranes to allow Ag atoms easy entry into the cells. As earlier researches reported, the internalization of NPs was size- and concentration-dependent [68, 69]. Additionally, the internalized AgNPs can induce cell death by causing macromolecular damage such as DNA fragmentation and protein denaturation [6, 70]. Further, AgNPs can enter into the cells through diffusion, endocytosis or the action of carrier proteins, and react with intracellular components, leading to the disintegration of cells and cell contents [46, 71]. Several studies have reported that after exposure of bacteria to NPs, cell wall damage and NPs were also found in passaged cells; these findings suggested that nanoparticles could be internalized and delivered to newly formed cells as a component of the cytoplasm [72, 73].

### Passible mechanism for toxicity

**Fig 8:**
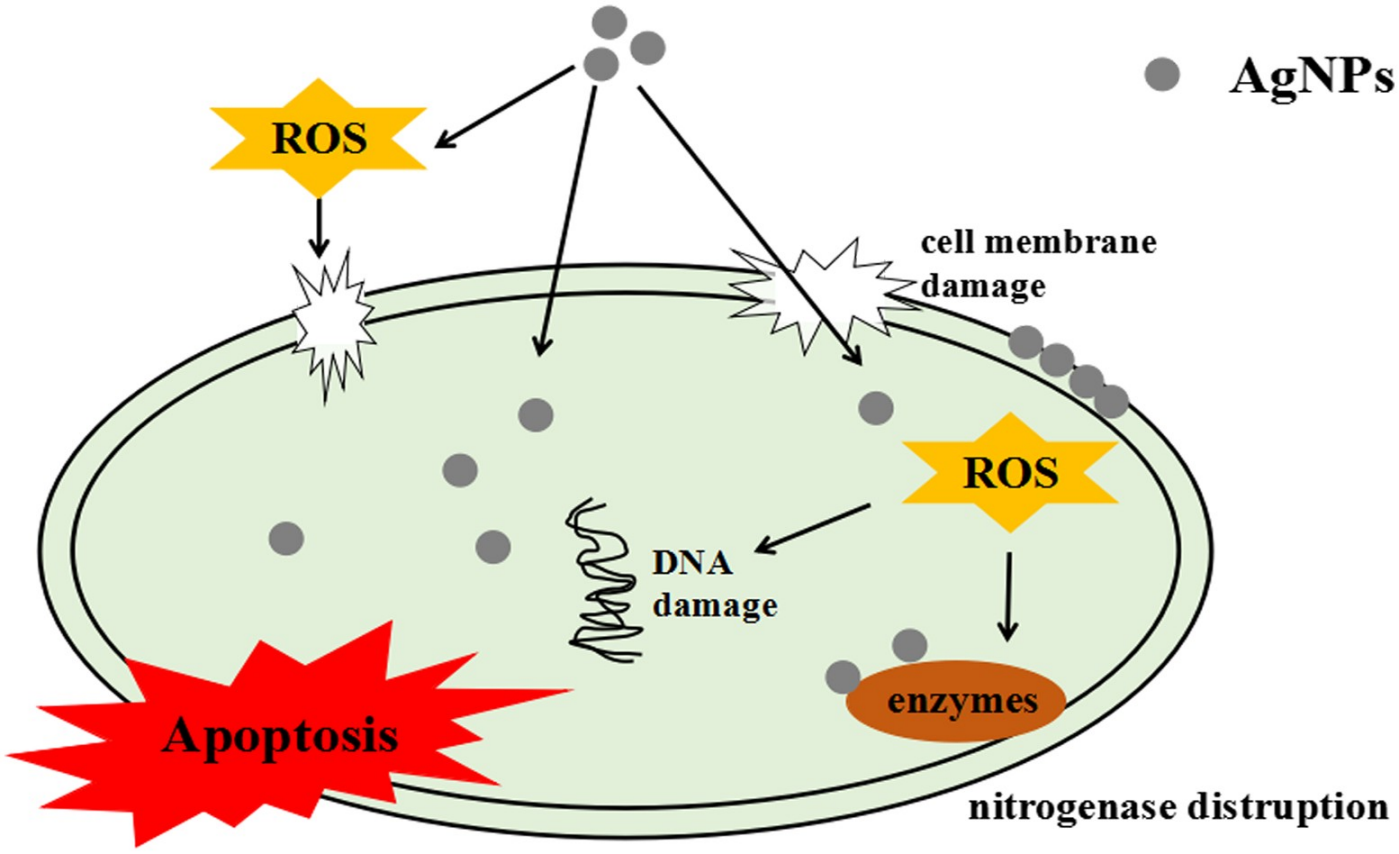
Possible toxicity mechanism for AgNPs in *A*.*vinelandii*.

The antibacterial activities of AgNPs were observed by a cell growth inhibition assay. Damages to cell morphology upon exposure to AgNPs are shown in Fig 3. Apoptosis of cells were determined by FCM. The impact of AgNPs on bacterial function was detected by enzyme activity and RT-qPCR assays. The intracellular ROS production was measured using the fluorescent dye H_2_DCFDA. Based on the results, we speculate that the antibacterial mechanisms of AgNPs (Fig 8) involve adherence to the surface of bacteria and destruction of the cell membrane structure. Smaller AgNPs more easily enter into the cell and react with sulfur-containing substances, such as DNA and proteins. These intracellular contents finally flow out along the pits in the damaged cell membrane; AgNPs can bind to sulfhydryl group (-SH) of enzymatic proteins in bacteria, inactivate the enzyme and inhibiting gene expression related to the enzyme; this ultimately interferes with the particular function of the bacteria. The presence of AgNPs also leads to the generation of ROS, which further damages cell structure and leads to bacterial apoptotic death [8, 13, 65]. We believe that the toxicity mechanism of AgNPs may be much more complicated than hypothesized. It may be that one or more mechanisms work together and even lead to more serious cell damage. Thus, detailed bactericidal activities still require more researches to verify.

## Conclusion

The possible mechanisms of AgNPs effects were explored and AgNPs were found to have significant inhibition on *A*. *vinelandii*. In addition, the toxicity of AgNPs was directly dose- and size- dependent. AgNPs inhibited the growth of bacteria and accelerated the cell apoptosis rate; it also affected the nitrogenase activity and expression of *nifH*. ROS detection and ESR analysis revealed that the presence of AgNPs could generate ROS and hydroxyl radicals. Electron microscopy combined with the distribution assay for AgNPs showed that AgNPs tend to attach to cell membrane, and damage the cells. At the same time, AgNPs could interact with *A*.*vinelandii* and could be internalizated in cells.

## References

[1] Tang B, Wang J, Xu S, Afrin T, Xu W, Sun L, et al Application of anisotropic silver nanoparticles: multifunctionalization of wool fabric. J Colloid Interface Sci. 2011;356(2):513–8. 10.1016/j.jcis.2011.01.054 . 21316697

[2] Chaloupka K, Malam Y, Seifalian AM. Nanosilver as a new generation of nanoproduct in biomedical applications. Trends Biotechnol. 2010;28(11):580–8. 10.1016/j.tibtech.2010.07.006 . 20724010

[3] Arnaout CL, Gunsch CK. Impacts of silver nanoparticle coating on the nitrification potential of Nitrosomonas europaea. Environ Sci Technol. 2012;46(10):5387–95. 10.1021/es204540z . 22533675

[4] Beddow J, Stolpe B, Cole P, Lead JR, Sapp M, Lyons BP, et al Effects of engineered silver nanoparticles on the growth and activity of ecologically important microbes. Environmental Microbiology Reports. 2014;6(5):448–58. 10.1111/1758-2229.12147 25646535

[5] Bryaskova R, Pencheva D, Nikolov S, Kantardjiev T. Synthesis and comparative study on the antimicrobial activity of hybrid materials based on silver nanoparticles (AgNps) stabilized by polyvinylpyrrolidone (PVP). J Chem Biol. 2011;4(4):185–91. 10.1007/s12154-011-0063-9 . 22837793 PMC3174283

[6] Riaz Ahmed KB, Nagy AM, Brown RP, Zhang Q, Malghan SG, Goering PL. Silver nanoparticles: Significance of physicochemical properties and assay interference on the interpretation of in vitro cytotoxicity studies. Toxicol In Vitro. 2017;38:179–92. 10.1016/j.tiv.2016.10.012 . 27816503

[7] Long YM, Hu LG, Yan XT, Zhao XC, Zhou QF, Cai Y, et al Surface ligand controls silver ion release of nanosilver and its antibacterial activity against Escherichia coli. Int J Nanomedicine. 2017;12:3193–206. 10.2147/IJN.S132327 . 28458540 PMC5402892

[8] Choi Y, Kim HA, Kim KW, Lee BT. Comparative toxicity of silver nanoparticles and silver ions to Escherichia coli. Journal of environmental sciences. 2018;66:50–60. 10.1016/j.jes.2017.04.028 . 29628108

[9] Farah MA, Ali MA, Chen SM, Li Y, Al-Hemaid FM, Abou-Tarboush FM, et al Silver nanoparticles synthesized from Adenium obesum leaf extract induced DNA damage, apoptosis and autophagy via generation of reactive oxygen species. Colloids and surfaces B, Biointerfaces. 2016;141:158–69. 10.1016/j.colsurfb.2016.01.027 . 26852099

[10] Seong M, Lee DG. Silver Nanoparticles Against Salmonella enterica Serotype Typhimurium: Role of Inner Membrane Dysfunction. Current microbiology. 2017;74(6):661–70. 10.1007/s00284-017-1235-9 . 28321528

[11] De Matteis V, Malvindi MA, Galeone A, Brunetti V, De Luca E, Kote S, et al Negligible particle-specific toxicity mechanism of silver nanoparticles: the role of Ag+ ion release in the cytosol. Nanomedicine. 2015;11(3):731–9. 10.1016/j.nano.2014.11.002 . 25546848

[12] Ahmed B, Hashmi A, Khan MS, Musarrat J. ROS mediated destruction of cell membrane, growth and biofilms of human bacterial pathogens by stable metallic AgNPs functionalized from bell pepper extract and quercetin. Advanced Powder Technology. 2018;29(7):1601–16. 10.1016/j.apt.2018.03.025

[13] Duran N, Duran M, de Jesus MB, Seabra AB, Favaro WJ, Nakazato G. Silver nanoparticles: A new view on mechanistic aspects on antimicrobial activity. Nanomedicine. 2016;12(3):789–99. 10.1016/j.nano.2015.11.016 . 26724539

[14] Shi T, Sun X, He Q-Y. Cytotoxicity of Silver Nanoparticles against Bacteria and Tumor Cells. Current Protein & Peptide Science. 2018;19(6):525–36. 10.2174/1389203718666161108092149 27829349

[15] Gliga AR, Skoglund S, Wallinder IO, Fadeel B, Karlsson HL. Size-dependent cytotoxicity of silver nanoparticles in human lung cells: the role of cellular uptake, agglomeration and Ag release. Particle and Fibre Toxicology. 2014;11 10.1186/1743-8977-11-11 24529161 PMC3933429

[16] Ema M, Okuda H, Gamo M, Honda K. A review of reproductive and developmental toxicity of silver nanoparticles in laboratory animals. Reprod Toxicol. 2017;67:149–64. 10.1016/j.reprotox.2017.01.005 . 28088501

[17] Liu W, Wu Y, Wang C, Li HC, Wang T, Liao CY, et al Impact of silver nanoparticles on human cells: effect of particle size. Nanotoxicology. 2010;4(3):319–30. 10.3109/17435390.2010.483745 . 20795913

[18] Zhang T, Wang L, Chen Q, Chen C. Cytotoxic potential of silver nanoparticles. Yonsei Med J. 2014;55(2):283–91. 10.3349/ymj.2014.55.2.283 . 24532494 PMC3936614

[19] Orlowski P, Zmigrodzka M, Tomaszewska E, Ranoszek-Soliwoda K, Czupryn M, Antos-Bielska M, et al Tannic acid-modified silver nanoparticles for wound healing: the importance of size. Int J Nanomedicine. 2018;13:991–1007. 10.2147/IJN.S154797 . 29497293 PMC5818815

[20] Nagpal P, Jafri S, Reddy MA, Das HK. Multiple chromosomes of Azotobacter vinelandii. Journal of bacteriology. 1989;171(6):3133–8. 10.1128/jb.171.6.3133-3138.1989 . 2785985 PMC210026

[21] Pajares S, Bohannan BJ. Ecology of Nitrogen Fixing, Nitrifying, and Denitrifying Microorganisms in Tropical Forest Soils. Front Microbiol. 2016;7:1045 10.3389/fmicb.2016.01045 . 27468277 PMC4932190

[22] Yu Z, Hao R, Zhang L, Zhu Y. Effects of TiO2, SiO2, Ag and CdTe/CdS quantum dots nanoparticles on toxicity of cadmium towards Chlamydomonas reinhardtii. Ecotoxicol Environ Saf. 2018;156:75–86. 10.1016/j.ecoenv.2018.03.007 . 29533210

[23] Han SO, New PB. Variation in Nitrogen Fixing Ability among Natural Isolates of Azospirillum. Microbial Ecology. 1998;36(2):193–201. 10.1007/s002489900106 9688781

[24] Wahab R, Khan F, Yang Yb, Hwang IH, Shin H-S, Ahmad J, et al Zinc oxide quantum dots: multifunctional candidates for arresting C2C12 cancer cells and their role towards caspase 3 and 7 genes. RSC Advances. 2016;6(31):26111–20. 10.1039/c5ra25668b

[25] Hakami O, Zhang Y, Banks CJ. Influence of aqueous environment on agglomeration and dissolution of thiol-functionalised mesoporous silica-coated magnetite nanoparticles. Environmental Science and Pollution Research. 2015;22(5):3257–64. 10.1007/s11356-014-3085-3 24898295

[26] Zhang C, Hu Z, Deng B. Silver nanoparticles in aquatic environments: Physiochemical behavior and antimicrobial mechanisms. Water Res. 2016;88:403–27. 10.1016/j.watres.2015.10.025 . 26519626

[27] Ellegaard-Jensen L, Jensen KA, Johansen A. Nano-silver induces dose-response effects on the nematode Caenorhabditis elegans. Ecotoxicol Environ Saf. 2012;80:216–23. 10.1016/j.ecoenv.2012.03.003 . 22475389

[28] Kaiser JP, Roesslein M, Diener L, Wichser A, Nowack B, Wick P. Cytotoxic effects of nanosilver are highly dependent on the chloride concentration and the presence of organic compounds in the cell culture media. J Nanobiotechnology. 2017;15(1):5 10.1186/s12951-016-0244-3 . 28061858 PMC5219688

[29] Reyes VC, Opot SO, Mahendra S. Planktonic and biofilm-grown nitrogen-cycling bacteria exhibit different susceptibilities to copper nanoparticles. Environ Toxicol Chem. 2015;34(4):887–97. 10.1002/etc.2867 . 25556815

[30] Gambino M, Marzano V, Villa F, Vitali A, Vannini C, Landini P, et al Effects of sublethal doses of silver nanoparticles on Bacillus subtilis planktonic and sessile cells. J Appl Microbiol. 2015;118(5):1103–15. 10.1111/jam.12779 . 25702880

[31] Choi O, Hu Z. Size dependent and reactive oxygen species related nanosilver toxicity to nitrifying bacteria. Environmental Science & Technology. 2008;42(12):4583–8. 10.1021/es703238h 18605590

[32] Braydich-Stolle LK, Lucas B, Schrand A, Murdock RC, Lee T, Schlager JJ, et al Silver nanoparticles disrupt GDNF/Fyn kinase signaling in spermatogonial stem cells. Toxicol Sci. 2010;116(2):577–89. 10.1093/toxsci/kfq148 . 20488942 PMC2905406

[33] Kim S, Ryu DY. Silver nanoparticle-induced oxidative stress, genotoxicity and apoptosis in cultured cells and animal tissues. J Appl Toxicol. 2013;33(2):78–89. 10.1002/jat.2792 . 22936301

[34] Beer C, Foldbjerg R, Hayashi Y, Sutherland DS, Autrup H. Toxicity of silver nanoparticles—nanoparticle or silver ion? Toxicol Lett. 2012;208(3):286–92. 10.1016/j.toxlet.2011.11.002 . 22101214

[35] Verma SK, Jha E, Sahoo B, Panda PK, Thirumurugan A, Parashar SKS, et al Mechanistic insight into the rapid one-step facile biofabrication of antibacterial silver nanoparticles from bacterial release and their biogenicity and concentration-dependent in vitro cytotoxicity to colon cells. RSC Advances. 2017;7(64):40034–45. 10.1039/c7ra05943d

[36] Yuan Z, Li J, Cui L, Xu B, Zhang H, Yu CP. Interaction of silver nanoparticles with pure nitrifying bacteria. Chemosphere. 2013;90(4):1404–11. 10.1016/j.chemosphere.2012.08.032 . 22985593

[37] Li WR, Xie XB, Shi QS, Zeng HY, Ou-Yang YS, Chen YB. Antibacterial activity and mechanism of silver nanoparticles on Escherichia coli. Appl Microbiol Biotechnol. 2010;85(4):1115–22. 10.1007/s00253-009-2159-5 . 19669753

[38] Michels C, Perazzoli S, H MS. Inhibition of an enriched culture of ammonia oxidizing bacteria by two different nanoparticles: Silver and magnetite. Sci Total Environ. 2017;586:995–1002. 10.1016/j.scitotenv.2017.02.080 . 28228236

[39] Giao NT, Limpiyakorn T, Kunapongkiti P, Thuptimdang P, Siripattanakul-Ratpukdi S. Influence of silver nanoparticles and liberated silver ions on nitrifying sludge: ammonia oxidation inhibitory kinetics and mechanism. Environ Sci Pollut Res Int. 2017;24(10):9229–40. 10.1007/s11356-017-8561-0 . 28224336

[40] Wang J, Shu K, Zhang L, Si Y. Effects of Silver Nanoparticles on Soil Microbial Communities and Bacterial Nitrification in Suburban Vegetable Soils. Pedosphere. 2017;27(3):482–90. 10.1016/s1002-0160(17)60344-8

[41] Choi O, Deng KK, Kim NJ, Ross L Jr., Surampalli RY, Hu Z. The inhibitory effects of silver nanoparticles, silver ions, and silver chloride colloids on microbial growth. Water Res. 2008;42(12):3066–74. 10.1016/j.watres.2008.02.021 . 18359055

[42] Chen Y, Chen J, Dong J, Jin Y. Comparing study of the effect of nanosized silicon dioxide and microsized silicon dioxide on fibrogenesis in rats. Toxicology and Industrial Health. 2004;20(1–5):21–7. 10.1191/0748233704th190oa 15807405

[43] Hakansson AP, Roche-Hakansson H, Mossberg AK, Svanborg C. Apoptosis-like death in bacteria induced by HAMLET, a human milk lipid-protein complex. PLoS One. 2011;6(3):e17717 10.1371/journal.pone.0017717 . 21423701 PMC3053380

[44] Yun J, Woo ER, Lee DG. Effect of isoquercitrin on membrane dynamics and apoptosis-like death in Escherichia coli. Biochim Biophys Acta Biomembr. 2018;1860(2):357–63. 10.1016/j.bbamem.2017.11.008 . 29155212

[45] Bao H, Yu X, Xu C, Li X, Li Z, Wei D, et al New toxicity mechanism of silver nanoparticles: promoting apoptosis and inhibiting proliferation. PLoS One. 2015;10(3):e0122535 10.1371/journal.pone.0122535 . 25822182 PMC4378976

[46] Wahab R, Dwivedi S, Khan F, Mishra YK, Hwang IH, Shin HS, et al Statistical analysis of gold nanoparticle-induced oxidative stress and apoptosis in myoblast (C2C12) cells. Colloids and surfaces B, Biointerfaces. 2014;123:664–72. 10.1016/j.colsurfb.2014.10.012 . 25456994

[47] Piao MJ, Kang KA, Lee IK, Kim HS, Kim S, Choi JY, et al Silver nanoparticles induce oxidative cell damage in human liver cells through inhibition of reduced glutathione and induction of mitochondria-involved apoptosis. Toxicol Lett. 2011;201(1):92–100. 10.1016/j.toxlet.2010.12.010 . 21182908

[48] Zapór L. Effects of silver nanoparticles of different sizes on cytotoxicity and oxygen metabolism disorders in both reproductive and respiratory system cells. Archives of Environmental Protection. 2016;42(4):32–47. 10.1515/aep-2016-0038

[49] Dixon R, Kahn D. Genetic regulation of biological nitrogen fixation. Nat Rev Microbiol. 2004;2(8):621–31. 10.1038/nrmicro954 . 15263897

[50] Haukka K, Lindstrom K, Young JP. Three phylogenetic groups of nodA and nifH genes in Sinorhizobium and Mesorhizobium isolates from leguminous trees growing in Africa and Latin America. Applied and environmental microbiology. 1998;64(2):419–26. . 9464375 10.1128/aem.64.2.419-426.1998 PMC106060

[51] Abd-Alla MH, Nafady NA, Khalaf DM. Assessment of silver nanoparticles contamination on faba bean-Rhizobium leguminosarum bv. viciae-Glomus aggregatum symbiosis: Implications for induction of autophagy process in root nodule. Agriculture, Ecosystems & Environment. 2016;218:163–77. 10.1016/j.agee.2015.11.022

[52] Zarate-Cruz GS, Zavaleta-Mancera HA, Alarcon A, Jimenez-Garcia LF. PHYTOTOXICITY OF ZnO NANOPARTICLES ON THE AQUATIC FERN Azolla filiculoides Lam. Agrociencia. 2016;50(6):677–91.

[53] Hou J, You G, Xu Y, Wang C, Wang P, Miao L, et al Antioxidant enzyme activities as biomarkers of fluvial biofilm to ZnO NPs ecotoxicity and the Integrated Biomarker Responses (IBR) assessment. Ecotoxicol Environ Saf. 2016;133:10–7. 10.1016/j.ecoenv.2016.06.014 . 27400059

[54] Li M, Yin JJ, Wamer WG, Lo YM. Mechanistic characterization of titanium dioxide nanoparticle-induced toxicity using electron spin resonance. J Food Drug Anal. 2014;22(1):76–85. 10.1016/j.jfda.2014.01.006 . 24673905 PMC9359148

[55] Katsumiti A, Thorley AJ, Arostegui I, Reip P, Valsami-Jones E, Tetley TD, et al Cytotoxicity and cellular mechanisms of toxicity of CuO NPs in mussel cells in vitro and comparative sensitivity with human cells. Toxicol In Vitro. 2018;48:146–58. 10.1016/j.tiv.2018.01.013 . 29408664

[56] He W, Zhou YT, Wamer WG, Boudreau MD, Yin JJ. Mechanisms of the pH dependent generation of hydroxyl radicals and oxygen induced by Ag nanoparticles. Biomaterials. 2012;33(30):7547–55. 10.1016/j.biomaterials.2012.06.076 . 22809647

[57] Li Y, Qin T, Ingle T, Yan J, He W, Yin JJ, et al Differential genotoxicity mechanisms of silver nanoparticles and silver ions. Arch Toxicol. 2017;91(1):509–19. 10.1007/s00204-016-1730-y . 27180073

[58] Wang E, Huang Y, Du Q, Sun Y. Silver nanoparticle induced toxicity to human sperm by increasing ROS(reactive oxygen species) production and DNA damage. Environ Toxicol Pharmacol. 2017;52:193–9. 10.1016/j.etap.2017.04.010 . 28433807

[59] Bagherzadeh Homaee M, Ehsanpour AA. Silver nanoparticles and silver ions: Oxidative stress responses and toxicity in potato (Solanum tuberosum L) grown in vitro. Horticulture, Environment, and Biotechnology. 2016;57(6):544–53. 10.1007/s13580-016-0083-z

[60] Vrcek IV, Zuntar I, Petlevski R, Pavicic I, Dutour Sikiric M, Curlin M, et al Comparison of in vitro toxicity of silver ions and silver nanoparticles on human hepatoma cells. Environmental toxicology. 2016;31(6):679–92. 10.1002/tox.22081 . 25448069

[61] Quinteros MA, Viviana CA, Onnainty R, Mary VS, Theumer MG, Granero GE, et al Biosynthesized silver nanoparticles: Decoding their mechanism of action in Staphylococcus aureus and Escherichia coli. The international journal of biochemistry & cell biology. 2018;104:87–93. 10.1016/j.biocel.2018.09.006 . 30243952

[62] Gurunathan S, Qasim M, Park C, Yoo H, Kim JH, Hong K. Cytotoxic Potential and Molecular Pathway Analysis of Silver Nanoparticles in Human Colon Cancer Cells HCT116. International journal of molecular sciences. 2018;19(8). 10.3390/ijms19082269 . 30072642 PMC6121495

[63] Gopinath V, Priyadarshini S, Al-Maleki AR, Alagiri M, Yahya R, Saravanan S, et al In vitro toxicity, apoptosis and antimicrobial effects of phyto-mediated copper oxide nanoparticles. RSC Advances. 2016;6(112):110986–95. 10.1039/c6ra13871c

[64] Geyik AG, Cecen F. Exposure of activated sludge to nanosilver and silver ion: Inhibitory effects and binding to the fractions of extracellular polymeric substances. Bioresour Technol. 2016;211:691–7. 10.1016/j.biortech.2016.03.157 . 27060244

[65] Das B, Dash SK, Mandal D, Ghosh T, Chattopadhyay S, Tripathy S, et al Green synthesized silver nanoparticles destroy multidrug resistant bacteria via reactive oxygen species mediated membrane damage. Arabian Journal of Chemistry. 2017;10(6):862–76. 10.1016/j.arabjc.2015.08.008

[66] Singh RP, Ramarao P. Cellular uptake, intracellular trafficking and cytotoxicity of silver nanoparticles. Toxicol Lett. 2012;213(2):249–59. 10.1016/j.toxlet.2012.07.009 . 22820426

[67] Bouallegui Y, Ben Younes R, Turki F, Mezni A, Oueslati R. Effect of exposure time, particle size and uptake pathways in immune cell lysosomal cytotoxicity of mussels exposed to silver nanoparticles. Drug Chem Toxicol. 2018;41(2):169–74. 10.1080/01480545.2017.1329317 . 28583008

[68] Hunt PR, Keltner Z, Gao X, Oldenburg SJ, Bushana P, Olejnik N, et al Bioactivity of nanosilver in Caenorhabditis elegans: Effects of size, coat, and shape. Toxicology reports. 2014;1:923–44. 10.1016/j.toxrep.2014.10.020 . 28962305 PMC5598322

[69] Zhao CM, Wang WX. Size-dependent uptake of silver nanoparticles in Daphnia magna. Environ Sci Technol. 2012;46(20):11345–51. 10.1021/es3014375 . 22974052

[70] Morones JR, Elechiguerra JL, Camacho A, Holt K, Kouri JB, Ramirez JT, et al The bactericidal effect of silver nanoparticles. Nanotechnology. 2005;16(10):2346–53. 10.1088/0957-4484/16/10/059 . 20818017

[71] Xia B, Chen B, Sun X, Qu K, Ma F, Du M. Interaction of TiO2 nanoparticles with the marine microalga Nitzschia closterium: growth inhibition, oxidative stress and internalization. Sci Total Environ. 2015;508:525–33. 10.1016/j.scitotenv.2014.11.066 . 25483108

[72] Wang L, Zheng H, Long Y, Gao M, Hao J, Du J, et al Rapid determination of the toxicity of quantum dots with luminous bacteria. J Hazard Mater. 2010;177(1–3):1134–7. 10.1016/j.jhazmat.2009.12.001 . 20056317

[73] Basu S, Jana S, Pande S, Pal T. Interaction of DNA bases with silver nanoparticles: assembly quantified through SPRS and SERS. J Colloid Interface Sci. 2008;321(2):288–93. 10.1016/j.jcis.2008.02.015 . 18346751

